# Polyethylene Glycol-Isophorone Diisocyanate Polyurethane Prepolymers Tailored Using MALDI MS

**DOI:** 10.3390/ma16020821

**Published:** 2023-01-14

**Authors:** Diana-Andreea Blaj, Alexandra-Diana Diaconu, Valeria Harabagiu, Cristian Peptu

**Affiliations:** “Petru Poni” Institute of Macromolecular Chemistry, Grigore Ghica Voda Alley, 41A, 700487 Iasi, Romania

**Keywords:** MALDI MS, MS/MS fragmentation, polyurethanes, polyethylene glycol, isophorone diisocyanate, kinetics

## Abstract

The reaction of diols with isocyanates, leading to mono-functional and di-functional prepolymers may be investigated using various characterization methods which show the overall conversion of isocyanate monomers. On the other hand, matrix-assisted laser desorption ionization mass spectrometry (MALDI MS) polymer characterization can be employed to identify the monomer units, the end-group functionalities, molecular weight averages, and to determine the copolymer sequence. Herein, we focus on prepolymer synthesis using isophorone diisocyanate (IPDI), a widely used diisocyanate for prepolymers preparation, especially in waterborne polyurethane materials. Thus, the reaction between polyethylene glycol diol and IPDI was in-depth investigated by mass spectrometry to determine the influence of the reaction parameters on the prepolymer’s structure. The relative content of the different functional oligomer species at given reaction times was determined in the reaction mixture. More specifically, the offline analysis revealed the influence of reaction parameters such as reaction temperature, the concentration of reactants, and the amount of dibutyltin dilaurate catalyst. The established MALDI MS analysis involved measurements of samples, first, directly collected from the reaction mixture and secondly, following derivatization with methanol. The obtained results revealed the effects of reaction parameters on the functionalization reaction with isocyanates, allowing to achieve a better reaction control.

## 1. Introduction

Polyurethanes are an essential class of polymeric materials obtained by the polyaddition reaction between a diisocyanate (hard segment) and a compound with multiple hydroxyl groups such as polyols (soft segment) [1]. The various chemical compositions of the precursors used for polyurethane synthesis, in terms of their chemical structure, render them versatile compounds applied for the preparation of a wide range of materials (flexible foams, rigid foams, chemical-resistant coatings, specialty adhesives, sealants, elastomers, biomaterials, etc.) [1].

Usually, a two-step reaction is involved in polyurethane synthesis: firstly, the formation of a prepolymer with terminal isocyanate groups (NCO), and secondly, the prepolymer coupling with a polyol. Prepolymerization reactions are very important in the formation of polyurethanes, influencing the properties of the final materials [2]. One of the most used polyols for obtaining polyurethane prepolymers is polyethylene glycol (PEG) and a considerable number of studies were performed on the reaction between PEG and diisocyanate compounds, as it is a key method for polyurethanes preparation [3,4,5,6,7,8,9,10,11,12,13,14,15].

Most diisocyanates used in polyurethane syntheses have similar isocyanate (NCO) groups in terms of reactivity towards hydroxyl groups (OH) (methylene diphenyl diisocyanate—MDI, hexamethylene diisocyanate—HMDI, etc.), and consequently, the prepolymerization reaction leads to subsequent chain extensions. On the other hand, isophorone diisocyanate (IPDI), an asymmetric aliphatic diisocyanate with different reactivity of the two NCO groups [16,17,18,19,20,21], represents an interesting candidate for prepolymer preparation. The significant difference in reactivity of the two isocyanate groups is essential in controlling the prepolymerization reaction [16]. The more reactive group reacts with the polyol while the remaining less reactive group can be involved in further polymerization reactions. This is an advantage in preventing the chain elongation and better controlling the reaction outcome.

In terms of reactivity, aliphatic diisocyanates can reach a level of reactivity close to aromatic ones, such as toluene diisocyanate (TDI), if an appropriate catalyst is used [1]. The use of organometallic catalysts (for example, dibutyltin dilaurate—DBTL), improves IPDI reactivity and selectivity [22,23].

In general, the reaction of diols with isocyanates will lead to mono-functional and di-functional prepolymers. The formation of these chemical species in the reaction mixture requires analytical methods that highlight the structural peculiarities at the molecular level of the products. The kinetics of the reaction between polyols and diisocyanates were investigated in a great number of studies using methods such as Fourier-transformed infrared spectroscopy (FTIR) [24,25,26,27,28,29,30], size exclusion chromatography (SEC) [31,32], differential scanning calorimetry (DSC) [33,34] and chemical titration of NCO functions [35,36]. In addition, such reactions were investigated by combining two or more analysis methods to obtain more accurate information regarding the reaction order for both components and to characterize the final products [37,38,39,40,41,42,43,44,45,46,47]. However, these methods are not capable of differentiating between mono and di-functional oligomers at a certain time, showing only the overall conversion. Moreover, the kinetics performed through classical spectroscopy techniques have a disadvantage in the analysis of functional oligomers because of the dilution of the target signal, e.g., the characteristic bands of isocyanate groups.

Lately, the reaction between various isocyanates and low molecular weight alcohols was also evaluated by electrospray ionization mass spectrometry (ESI MS) and validated by liquid chromatography with UV spectroscopy detection showing an excellent agreement between the two methods [48]. Thus, ESI MS allowed a comparative evaluation of the isocyanate reactivities for specific temperature and concentration reaction conditions. On the other hand, matrix-assisted laser desorption/ionization mass spectrometry (MALDI MS) can be employed to accurately determine the structure of prepolymers (oligomers with molecular weight averages in the region 2000 g/mol) [49,50]. For example, Nagy et al. [49] evaluated the mixture content, for reactions of MDI with different polyols, using the MALDI MS method. Monitoring the minute changes in the reaction mixture, the authors provided not only qualitative information regarding the structural changes but, more importantly, the MS reaction kinetics gave semi-quantitative information about the content of the existing species and allowed determination of specific reactivities in the prepolymer synthesis.

In general, MALDI MS can be used to identify the monomer units, the end-group functionalities, molecular weights, and their distribution, and to determine the copolymer sequence [51,52,53,54]. For example, Beldi et al. [55] combined MALDI MS and NMR analyses to characterize various linear and cyclic polyurethanes based on starch-derived diols and two different diisocyanates (MDI, HMDI). The reaction between polypropylene glycol (PPG) and IPDI was followed by MALDI MS, the mass spectra showing the formation of the mono-functional prepolymer which was further transformed into the di-functional prepolymer by the end of the reaction time [50]. In a recent study, MALDI MS was used to analyze poly(ε-caprolactone)-diol-HMDI prepolymers and also reacted products between prepolymer and sucrose in the early crosslinking stage [56]. More recently, MALDI MS was successfully employed to investigate the reactive di-functional prepolymer formed from polyethylene glycol (PEG) and IPDI [15].

Although polyols addition reactions to e.g., MDI were thoroughly investigated demonstrating the utility of the MALDI MS method [49], the influence of the specific reaction conditions such as concentration, temperature, and catalyst content, on the outcome of prepolymer synthesis, remains uncharted. Herein, we focus on the prepolymer synthesis using IPDI, a widely used diisocyanate for polyurethane materials [1], especially due to the high difference in the reactivity of the isocyanate groups. Thus, the PEG-diol-IPDI reaction was in-depth investigated by MALDI MS to determine the influence of the reaction parameters on the prepolymers structure and reaction mixture content at specific reaction times. More specifically, we investigated the reaction evolution according to the offline MS analysis to determine the state of each component (unreacted PEG, IPDI mono-functional PEG, and IPDI di-functional PEG).

## 2. Materials and Methods

Materials: Polyethylene glycol (PEG, *Mn* = 2000 g/mol) was purchased from Sigma Aldrich (Saint Louis, MO, USA). *N,N*-dimethylformamide (DMF, Sigma Aldrich, Saint Louis, MO, USA), and tetrahydrofuran (THF, VWR International, Vienna, Austria) were distilled under a vacuum and kept over activated 3Å molecular sieves for 3 days before use. Isophorone diisocyanate (IPDI) and dibutyltin dilaurate (DBTL) were acquired from Sigma Aldrich (Saint Louis, MO, USA) and used as received. For MALDI MS analysis, the matrices (*trans*-2-(3-(4-*tert*-butylphenyl)-2-methyl-2-propenylidene)malononitrile—DCTB or 2,5-dihydroxybenzoic acid—DHB) and sodium iodide (NaI) were purchased from Sigma Aldrich (Saint Louis, MO, USA), while methanol was from VWR International (Vienna, Austria).

Synthesis: Polyethylene glycol was introduced in a two-neck round bottom flask equipped with a rubber stopcock, a vacuum connector, and a mechanical stirrer. The flask containing PEG was placed on a heating plate with an oil bath (at 100 °C) and magnetic stirring (300 rpm) and kept for 3 h under vacuum (at 10^–2^ mbar) to remove the water traces. To obtain urethane prepolymers, dry DMF was added over the dried PEG under the protection of Ar gas and left to homogenize for a few minutes at room temperature. After homogenization, IPDI was added in a PEG/IPDI molar ratio of 1/2. Further, established amounts of DBTL were added to the reaction mixture according to Table 1. Samples (20 μL) were collected from all reaction mixtures at specific times up to 4 h using a gas-tight syringe and further analyzed using MALDI MS.

Mass Spectrometry: MALDI MS analysis was performed using a RapifleX MALDI TOF TOF MS instrument (Bruker, Bremen, Germany). FlexControl 4.0 and FlexAnalysis 4.0 software (Bruker) were used to control the instrument and process the MS and MS/MS spectra.

The samples for MS analysis were prepared as follows:

- Reactive samples: Samples from the reaction mixture were rapidly introduced in dry THF and mixed using a Vortex-Genie 2 device. The DCTB matrix was prepared in THF at a concentration of 10 mg/mL, while the NaI concentration in THF was 5 mg/mL.
- Quenched samples: Samples from the reaction mixture were introduced in MeOH for 24 h to quench the reactive NCO groups. The DHB matrix and NaI solutions were prepared in methanol at concentrations of 20 mg/mL, and, respectively, 5 mg/mL.

The samples were applied on the MALDI steel plate using the dried droplet method: 20 μL of matrix solution was mixed with 2 μL of NaI and 2 μL of sample solution and 1 μL from this mixture was deposited on the ground steel plate. The spectra were acquired in the positive reflectron mode, and the laser ionization power was adjusted slightly above the threshold to produce consistent MS signals. 18000 spectra were collected from different regions of the spot for each sample. The obtained MALDI MS spectra were further used to follow the reaction kinetics. The MS calibration was performed using poly(ethylene glycol) standards applied to the MALDI MS target.

The MS/MS fragmentation experiments were performed in LIFT mode using a Bruker standard fragmentation method. The full isotopic profile of the parent ion was isolated.

The number average molecular weight was determined by MALDI MS using the following formula:

(1) $$M_{n}=\frac{\sum_{i}^{n}I_{i}\times m_{i}}{\sum_{i}^{n}I_{i}}$$

where: *I_i_*—monoisotopic peak intensity corresponding to the *m_i_*; *m_i_*—*m*/*z* value of the corresponding *i* peak, with *z =* 1.

Peak integration was performed in the FlexAnalysis software (Bruker) using the following parameters: snap peak detection algorithm, signal-to-noise threshold = 6, and the maximal number of peaks = 100. These settings automatically provide MS peak lists containing the monoisotopic peak from the isotopic cluster. In the case of overlapping isotopic clusters, the monoisotopic peaks were manually assigned.

The intensities from the mass spectra of the products obtained after the quenching reaction were used to calculate the fractional content for the analyzed mixture (series *P*0 (initial PEG), *P*1 (mono-functional PEG), and *P*2 (di-functional PEG)) using the following formulae:

(2) $$X_{P0}=\frac{\sum I_{P0i}}{\sum (I_{P0i}+I_{P1i}+I_{P2i})}$$

(3) $$X_{P1}=\frac{\sum I_{P1i}}{\sum (I_{P0i}+I_{P1i}+I_{P2i})}$$

(4) $$X_{P2}=\frac{\sum I_{P2i}}{\sum (I_{P0i}+I_{P1i}+I_{P2i})}$$

where: *I_P_*_0*i*_, *I_P_*_1*i*_, and *I_P_*_2*i*_—the MALDI-MS peaks intensities corresponding to the respective oligomers’ series *P*0, *P*1, and *P*2.

In principle, the relative intensities of the products with different structures and their concentration in the analyzed mixture are not directly proportional, and this situation is specific to any analyzed molecule. The proportionality relationship may be established using correction factors as described in the following formulae:

(5) $$I_{P0i}=f_{P0}\times [P0i]$$

(6) $$I_{P1i}=f_{P1}\times [P1i]$$

(7) $$I_{P2i}=f_{P2}\times [P2i]$$

where *f_P_*_0_, *f_P_*_1_ and *f_P_*_2_ are the MALDI-MS response factors for series *P*0, *P*1 and *P*2, respectively.

The molar fractions, *X_P_*_0_, *X_P_*_1_, and *X_P_*_2_ for *P*0, *P*1, and *P*2 peaks series can be given using Equations (5)–(7):

(8) $$X_{P0}=\frac{\frac{\sum I_{P0i}}{f_{P0}}}{\frac{\sum I_{P0i}}{f_{P0}}+\frac{\sum I_{P1i}}{f_{P1}}+\frac{\sum I_{P2i}}{f_{P2}}}=\frac{\sum I_{P0i}}{\sum I_{P0i}+(\frac{f_{P0}}{f_{P1}})\sum I_{P1i}+(\frac{f_{P0}}{f_{P2}})\sum I_{P2i}}$$

(9) $$X_{P1}=\frac{\frac{\sum I_{P1i}}{f_{P1}}}{\frac{\sum I_{P0i}}{f_{P0}}+\frac{\sum I_{P1i}}{f_{P1}}+\frac{\sum I_{P2i}}{f_{P2}}}=\frac{(\frac{f_{P0}}{f_{P1}})\sum I_{P1i}}{\sum I_{P0i}+(\frac{f_{P0}}{f_{P1}})\sum I_{P1i}+(\frac{f_{P0}}{f_{P2}})\sum I_{P2i}}$$

(10) $$X_{P2}=\frac{\frac{\sum I_{P2i}}{f_{P2}}}{\frac{\sum I_{P0i}}{f_{P0}}+\frac{\sum I_{P1i}}{f_{P1}}+\frac{\sum I_{P2i}}{f_{P2}}}=\frac{(\frac{f_{P0}}{f_{P2}})\sum I_{P2i}}{\sum I_{P0i}+(\frac{f_{P0}}{f_{P1}})\sum I_{P1i}+(\frac{f_{P0}}{f_{P2}})\sum I_{P2i}}$$

In the work of Nagy et al. [49], the ratio between the response factors for initial and NCO functional oligodiols (PPG, PTHF, and PCL) was experimentally found equal to unity, so the Equations (2)–(4) may be directly used to calculate the molar fraction for each series (*P*0, *P*1, and *P*2) implicated in the reaction system.

Evaluation of the apparent reaction rate (*k_app_*) by MALDI MS:

The kinetic analysis can also be evaluated by an apparent reaction rate calculated from the conversion of the *P*0 species assuming a process of transformation from *P*0 to *P*2 which is characterized by an apparent reaction rate (*k_app_*). The value of *k_app_* may be expressed based on the consumption of *P*0 species and the appearance of *P*2 species. In principle, *P*0 species are transformed into *P*1 species and then into *P*2 species, but this sequence of reactions is deliberately ignored. Then, *k_app_* can be determined from the slope of *ln*([*P*0]_0_/[*P*0]*_t_*) evolution in time, where [*P*0*]_t_ = [P*0]_0_ − [*P*2*]_t_*, [*P*0]_0_—initial PEG concentration, [*P*0*]_t_*—concentration of unreacted PEG at a certain time, and [*P*2*]_t_*—di-functional PEG at a certain time.

(11) $$k_{app}=\frac{d(ln\frac{{\left[P0\right]}_{0}}{{\left[P0\right]}_{0}-{\left[P2\right]}_{t}})}{dt}$$

Taking into considerations Equations (2)–(10), molar fractions determined by MALDI MS are further used for the calculations of *k_app_*, as follows:

(12) $$k_{app}=\frac{d(ln\frac{{\left(X_{P0}\right)}_{o}}{{\left(X_{P0}\right)}_{0}-{\left(X_{P2}\right)}_{t}})}{dt}$$

where (*X_P_*_0_)_0_—is the initial molar fraction of PEG, and (*X_P_*_2_)_0_—the molar fraction of di-functional PEG at time *t*.

## 3. Results and Discussion

**Scheme 1:**
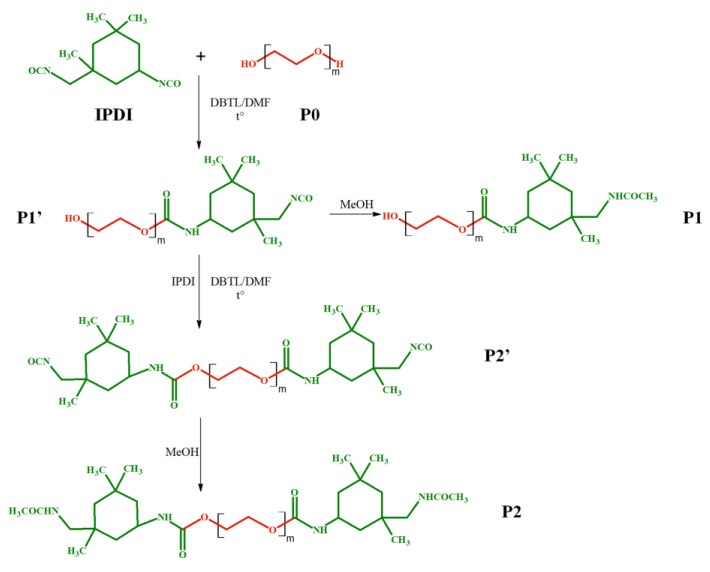
Schematic representation of the di-functional product formation after interaction of PEG and two molecules of IPDI (*P*2), with an intermediate mono-functional product formation (*P*1).

The polyaddition reaction between IPDI and PEG presented in Scheme 1, being given its practical importance, was chosen to demonstrate the possibility of optimization using MALDI MS-assisted kinetics. Thus, several reaction parameters were varied, such as reaction temperature (25, 35, and 50 °C), the concentration of reactants in DMF, and the amount of catalyst (Table 1) while the molar ratio between PEG and IPDI was kept constant, at 1/2. Fractions were collected at specific time intervals from the reaction mixture and analyzed by MALDI MS in specific conditions as further described.

As shown in Scheme 1, the formation of the di-functional PEG (*P*2′ in Scheme 1) involves firstly a polyaddition reaction step between PEG (type of chains noted *P*0) and IPDI with the formation of the *P*1′ species, the mono-functional PEG—Scheme 1. Subsequently, the *P*1′ chains react on their turn with IPDI to form the di-functional compound *P*2. In this context, the MALDI MS method sheds light on the relative ratio of the different functionalized PEG chains at a certain reaction time, thus reflecting the influence of the varied reaction parameters.

A relatively common problem encountered when using MALDI MS for such a purpose is represented by the biased quantitative response when dealing with polymer mixtures [51]. Therefore, an important issue concerning our study is related to the employed MALDI MS matrix type, among others. For polyurethane analysis, dithranol [50,52], α-cyano-4-hydroxycinnamic acid (α-CHCA) [57,58], 2,5-dihydroxy benzoic acid (DHB) [49,56,59], and *trans*-2-(3-(4-*tert*-butylphenyl)-2-methyl-2-propenylidene)malononitrile (DCTB) [15] matrices are most often employed. Recently, DCTB has been proven to be a good matrix for MS analysis of polymers with different structures or molecular weights, including PEG [60].

In principle, various MALDI MS analyzed species may present a characteristic response factor, translated in MALDI MS intensity of the corresponding peaks. Relative MS peaks intensities do not necessarily correlate with the relative content in the polymer mixture. Generally, this response factor is connected with different solubility of the samples, sample matrix interactions, molecular weight averages and dispersity, analysis conditions, etc. [51]. A previous study [49], on PPG, poly(ε-caprolactone), and polytetrahydrofuran-based urethane prepolymers, employed alcohol derivatization of the isocyanate end-functions and used DHB as the MALDI MS matrix. They showed that, for rather low molecular weight chains in the region of 2000 g/mol, the response factor between original and NCO-modified chains is not affected, thus allowing semi-quantitative measurements.

**Figure 1:**
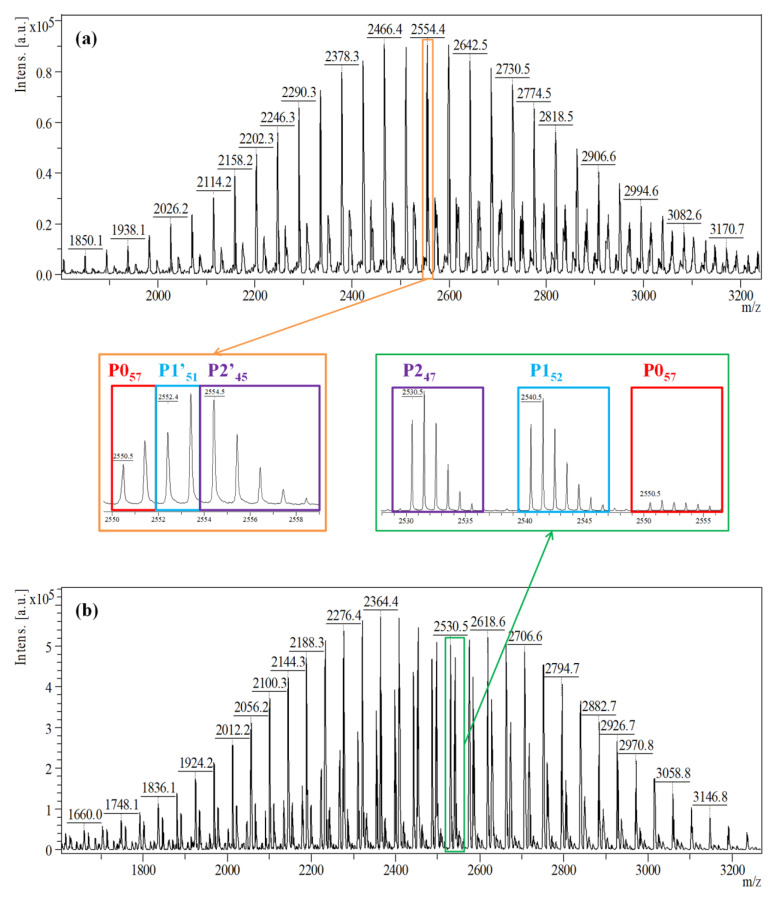
MALDI MS spectra of (**a**) the reactive products, and (**b**) the quenched products of the reaction mixture obtained in reaction #2 at 15 min. The numbers in the subscript from the enlarged area refer to the number of ethylene oxide units.

In the current study, we aim to establish the differences between the two methods of sample preparation for MALDI MS measurement, first by directly analyzing the prepolymer structures without quenching and using DCTB matrix, and second by performing methanol derivatization and MS analysis using DHB matrix. The MALDI MS spectrum of the reactive samples collected from reaction #2 (Table 1), in Figure 1a, reveals one main Gaussian distribution corresponding to three distinct overlapping populations (unreacted PEG, mono-, and di-functional PEG noted as *P*0, *P*1′, and *P*2′, respectively), as highlighted. The sequence of the main series corresponds to the PEG oligomer (Δ*m*/*z* = 44 Da—the mass of the ethylene oxide monomer unit) having as end-chains IPDI functions.

This assignment of the spectrum peaks is based on the equations presented in Table 2. Thus, the highlighted peak from Figure 1a corresponds to the sodium adduct of an IPDI di-functional PEG having *n* = 45 ethylene oxide monomer units (marked as *P*2_45_). However, in the immediate vicinity and overlapping, two peaks—*P*0_57_ and *P*1′_51_ belonging to another two series, may be also observed and they were assigned as presented in Table 2. Because of the overlapping of the full isotopic profile, the peaks from the *P*0, *P*1′, and *P*2′ series are hardly distinguished from one another, and the semi-quantitative analysis of reactive products fails.

On the other hand, the MALDI MS spectrum registered by MeOH quenching of the NCO functions in the collected samples (Figure 1b) shows a remarkable difference. The peaks corresponding to the mono-, and di-functional series are up-shifted by 32 Da (noted *P*1), and, respectively, 64 Da (noted *P*2), following the addition of methanol molecules. Thus, MeOH derivatization renders each population easily recognizable and more importantly, quantifiable.

**Figure 2:**
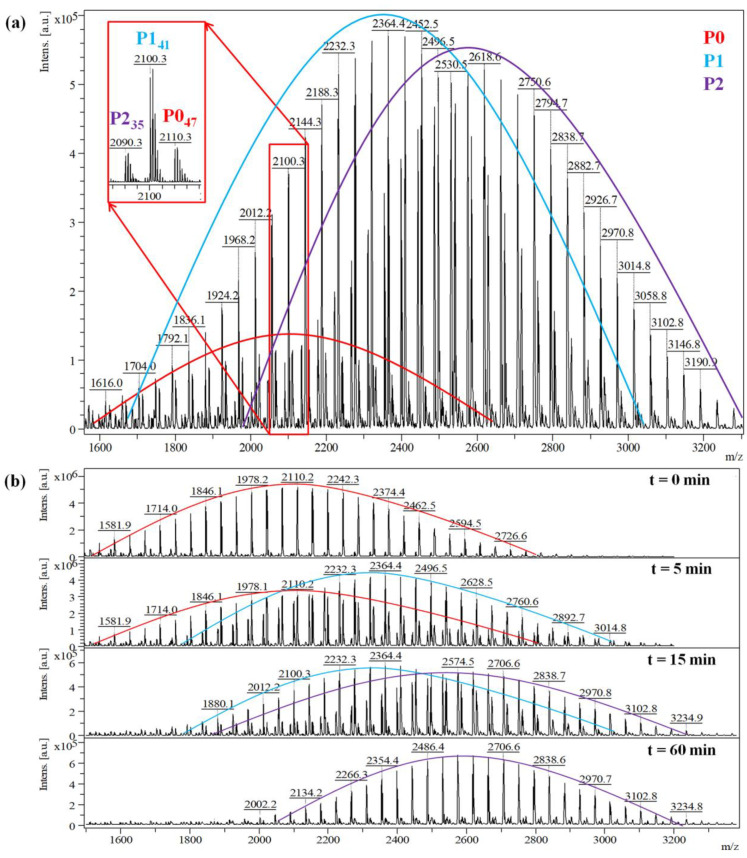
MALDI MS spectrum of the quenched reaction mixture obtained in reaction #2 at (**a**) 15 min, and (**b**) at different reaction times, highlighting the three existing populations in the system (unreacted PEG*—P*0, mono-, and di-functional PEG*—P*1, respectively *P*2).

Therefore, the MALDI MS spectrum of the quenched fraction collected after 15 min of reaction time (#2 synthesis), shows three main series corresponding to the *P*0, *P*1, and *P*2 species (Figure 2a). Each peak series is described using the equations given in Table 3. The highlighted peak species presented as an example in Figure 2a, are assigned as follows: the peak from the *P*0 series at *m*/*z* = 2110 g/mol corresponds to 47 units of ethylene oxide, while the peak from the *P*1 series at *m*/*z* = 2100 g/mol and the peak from the *P*2 series at *m*/*z* = 2090 g/mol corresponds to 41 and 35 units of ethylene oxide, respectively. Moreover, the spectra presented in Figure 2b, obtained from the timely collected samples, reveal the evolution of the three PEG peak series, from *P*0 to *P*2. These spectra may be further used to calculate the specific content of the reaction mixture, expressed as molar fractions.

Moreover, MS/MS fragmentation studies confirmed the structures of mono- and di-functional PEG chains, as given in the supplementary information (Figures S1 and S2, Schemes S1 and S2).

**Figure 3:**
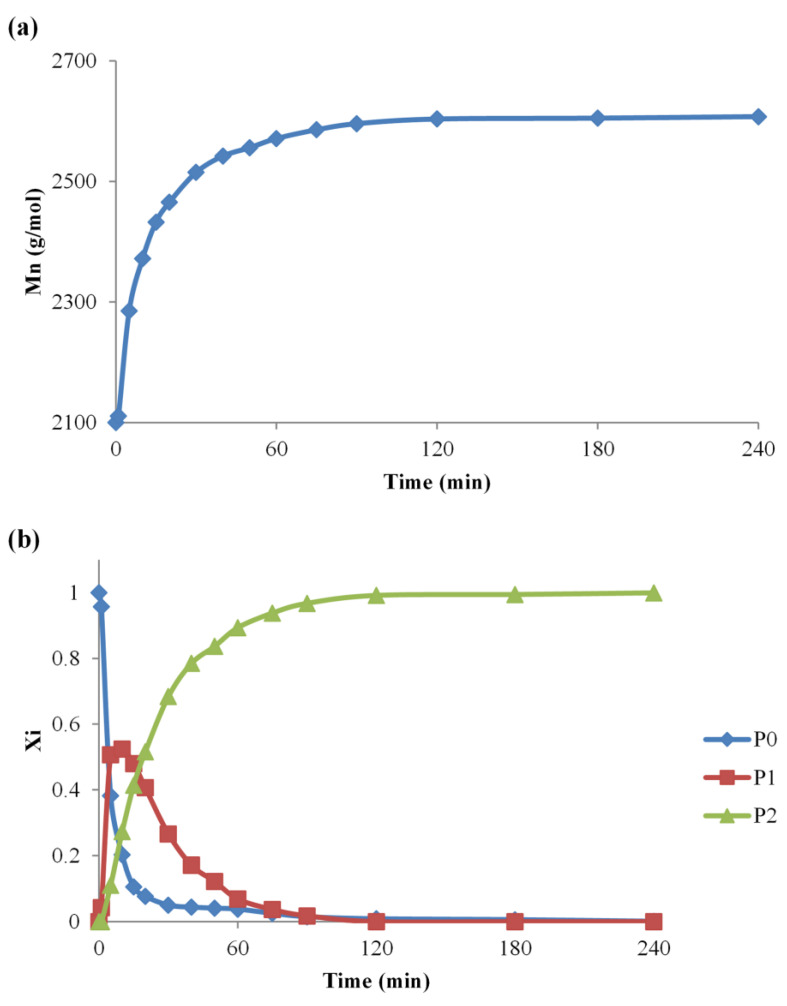
The *Mn* evolution for the #2 reaction—(**a**) overall *Mn* values plot and (**b**) separate plot of *P*0, *P*1, and *P*2 species.

The utility of the data obtained from MALDI MS analysis is exemplified by monitoring the chemical nature and the relative content of the PEG functionalization (#2 synthesis) at specific reaction times (Figure 3a). The evolution of the PEG functionalization reaction may be followed by simply taking into consideration all the peaks from the MALDI MS spectrum to calculate the average *Mn* of each collected sample, using equation (1). The plot of the overall *Mn* values against time reveals the moment when the *Mn* increase of the original PEG reaches the theoretical *Mn* of the di-functional PEG, as observed in Figure 3a. The observed overall increase from 2100 g/mol to 2608 g/mol, corresponds to the attachment of two IPDI and two MeOH molecules, originating from the quenched reaction mixture Δm = (2 × 222 + 2 × 32) g/mol = 508 g/mol. Such an approach is useful when analyzing and comparing reaction systems that reach full conversion of the reactive species. However, the specific reaction conditions may lead to a diminished conversion, and, in such situations, a more precise approach would consider a deconvolution of the overall *Mn* increase (Figure 3b), to observe the evolution of each type of chain and to clearly evidence the relative amount of *P*1 and *P*2 species.

The analysis of the quenched reaction mixtures (Figure 3b) allows the determination of the evolution of each type of PEG population. As expected, the reaction of PEG with IPDI leads to a fast formation of *P*1 species followed by a decrease as they evolve rapidly into *P*2 species, by reacting with another IPDI molecule. This approach shows accurately the evolution of the system, and it was further used to observe the influence of temperature, reactants concentration, and catalyst amount over the PEG-IPDI reaction.

- Temperature influence—MALDI MS

The temperature effect on the addition of model primary OH functions (n-butanol) to IPDI, catalyzed by DBTL, may be resumed to the increase in the system reactivity together with the reduction in the reaction selectivity to the secondary NCO group [61], as demonstrated through gas chromatography studies. The recommended temperature range for the DBTL-catalyzed reactions is 40–60 °C, but temperatures of 35 °C provide also good control over the resulting prepolymer, as shown by later studies [44].

**Figure 4:**
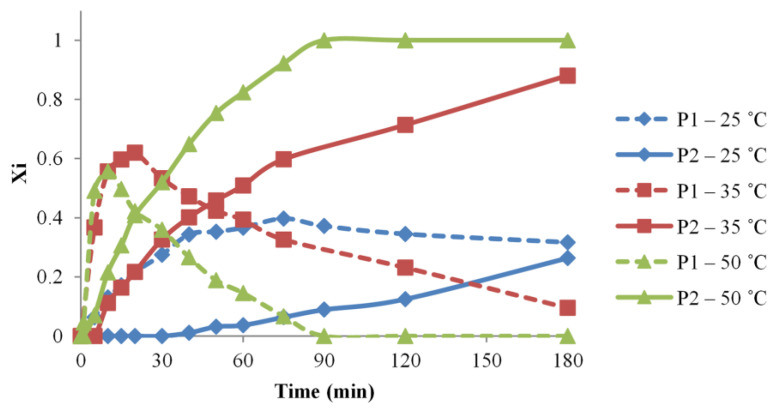
Temperature influence over the urethane formation determined by MALDI MS—variations of *P*1 and *P*2 relative molar fractions in the function of time (#1, 5, 6 reactions).

Thus, in the current study, the temperatures of 25, 35, and 50 °C, respectively, were considered, aiming to describe the reaction system, qualitatively and quantitatively. In Figure 4, the evolution of the relative content in *P*1 and *P*2 species is represented according to the reaction temperature. The formation of *P*1 species for the 35 and 50 °C reactions undergoes almost as fast, but because of a better consumption of *P*1 and transformation into *P*2 for the highest tested temperature, *P*1 species reach a higher relative content in the reaction carried at 35 °C. However, the reaction performed at 25 °C leads to a low relative amount of *P*1 species.

The differences induced by temperature variation are more clearly observed in the evolution of the *P*2 species. Thus, both the 35 and 50 °C temperatures allow reaching the full transformation into *P*2 species, however, with a significant time difference of about 150 min. The evaluation of the lower reaction temperature revealed that the *P*2 amount is lower, and, in the employed conditions, the necessary reaction time for completion should be excessively long. A clear difference between syntheses #1, 5, and 6 may be ascribed by calculating the specific apparent reaction rates based on the evolution of *P*2 species, as calculated using Equation (12). Thus, the calculated *k_app_* values are increasing with the reaction temperatures (Table 1). Moreover, the *k_app_* variation with the temperature was found linear demonstrating that similar processes take place in the reaction mixtures at all the employed temperature conditions.

Our experiments demonstrate that MALDI MS kinetics reflect accurately the relative amounts of *P*1 and *P*2 species, without making a clear difference in the reaction selectivity of the IPDI isocyanate functions. However, it is expected that the selectivity is better for lower reaction temperatures.

- Concentration influence

Prepolymerization reactions are performed in bulk, but solvents may be used for better control, e.g. to prevent chain extension reactions. Generally, for diluted systems, the concentration increase should speed up the monomer conversion [20] and, ideally, this parameter may be set to ensure the formation of the prepolymer without the interference of secondary processes. Particularly, in the case of IPDI, the difference in reactivity between the primary and secondary NCO functions allows a better success rate in the obtention of the di-functional prepolymers. Nevertheless, the MALDI MS kinetics may allow the differentiation between overall NCO functions conversion to urethanes and the actual structure of the reaction products throughout prepolymerization [49].

**Figure 5:**
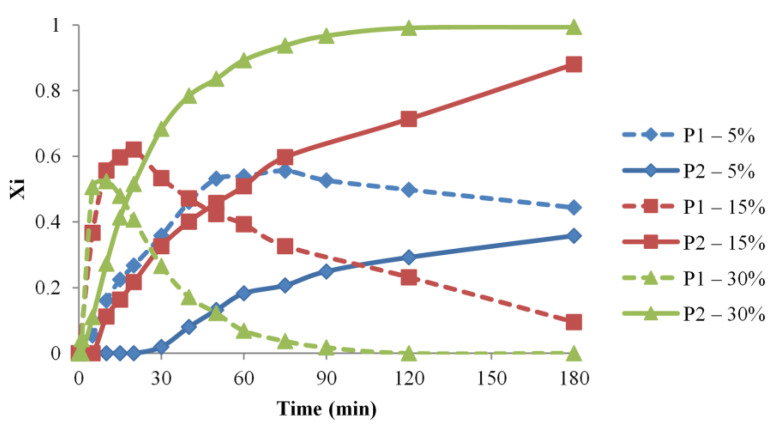
*P*1 and *P*2 relative molar fractions evolution for various concentration conditions (#1, 2, 3).

The concentration influence on the PEG-IPDI reaction has been studied herein for three different concentrations, in DMF at 35 °C, and the monitoring of the reaction mixture contents is presented in Figure 5. Thus, the performed MALDI MS kinetics revealed that there are significant differences concerning the appearance of both *P*1 and *P*2 species, as the overall concentration is increased from 5 to 30% wt.

The time frame for the considered syntheses, 180 min, allowed the full conversion into *P*2 species only for the highest concentration (30% wt). Moreover, there may be observed that the formation of *P*1 species is faster at higher concentrations, and their consumption rate, due to the formation of *P*2 species, is similarly dependent on concentration. Thus, the calculated apparent reaction rate values for syntheses #1–3 are correlated with the concentration increase.

Therefore, the performed MS analysis reflects the fact that playing with the concentration parameter allows controlling the amounts of NCO-functionalized OH groups in the reaction mixture. This may be significantly important for experiments that are aiming to minimize the propensity of addition reactions, e.g., for waterborne polyurethanes. Moreover, the control of the reactions between isocyanates and polyols with a higher number of OH groups may greatly benefit from a better understanding of the concentration effects on the level of functionalization [62].

- Catalyst influence

A large number of publications describe the amount of added DBTL catalyst in prepolymer synthesis reactions as “one or two drops”, based on the fact that such an amount is generally enough for the reaction completion. However, for a reaction system in tetrahydrofuran solvent, Burel et al. [30] demonstrated that the reaction rate presents a linear dependence on DBTL molar concentration up to a limit of 2 × 10^−3^ mol L^−1^. Moreover, a high concentration of DBTL affects the reaction kinetics by the appearance of complexes or aggregates made by the catalyst with members of the system. In addition, the increase in the concentration of the catalyst leads to a decrease in the addition selectivity of the IPDI isocyanate groups [30]. Therefore, the amount of catalyst employed in the current study for the tested parameters (concentration and temperature) is the equivalent of 2.5 × 10^−3^ mol L^−1^, a value slightly higher than that mentioned in the literature studies. The influence of the DBTL amount in prepolymerization reactions was also studied in the case of polybutadiene polyols containing thioether (PHPBT) with IPDI, quantifying the results by the NCO chemical titration method [44]. The study revealed that the isocyanates residual content decreased with the increase in DBTL amount. However, by increasing the DBTL amount over a certain limit, the NCO residual content and the reaction rate do not show significant changes.

**Figure 6:**
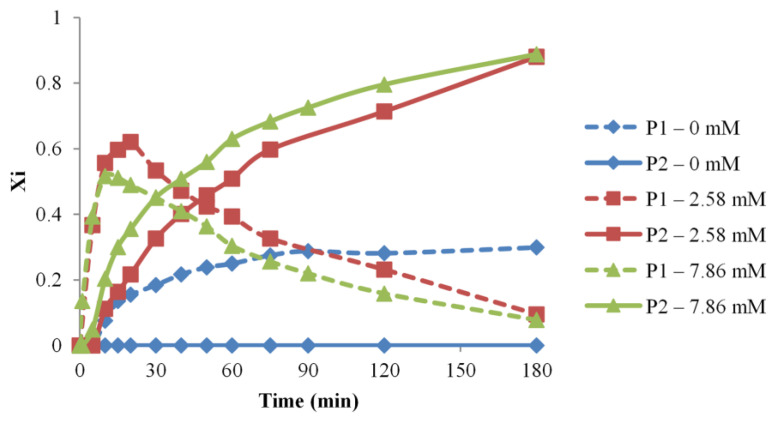
Evolution of the NCO-functional PEG populations (*P*1 and *P*2 relative molar fractions) versus time for different amounts of catalyst (reaction #1, 4, 7).

The influence of the catalyst amount was tested for three different reaction systems, containing 7.8 × 10^−3^ M (#4), 2.5 × 10^−3^ M (#1), and no catalysts (#7), respectively. The catalyst effect, monitored by MALDI MS as depicted in Figure 6, revealed a change in the *k_app_* values specific to the overall transformation from *P*0 to *P*2 species, from (1.13 × 10^−2^ for #1, 1.59 × 10^−2^ for #4), thus, indicating a significant increase in the system reactivity. On the other hand, the reaction performed without a catalyst does not lead to the formation of *P*2 species, denoting a very low overall reactivity in the system.

## 4. Conclusions

The employed MALDI MS method to directly characterize the PEG-IPDI isocyanate-terminated reactive oligomers was not able to differentiate the mixture components. However, the method that involves methanol derivatization of the reactive isocyanate oligomers allows quantification of the PEG species in the collected mass spectra. The MS/MS experiments were further used to confirm the structural assignments. MS monitorization of the timely collected samples allowed us to follow independently the mono- and di-functional PEG. The reactions were evaluated considering the formation of di-functional PEG species and apparent reaction rates were calculated for specific synthesis parameters based on the MS-determined molar fractions. Thus, mass spectrometry evaluation revealed the effects of reaction temperature, the concentration of the reagents, and catalyst amounts, demonstrating the utility of the employed method. Based on the acquired data optimal conditions for the preparation of the oligomers may be further envisaged with prospects to extend the current approach to more complex systems based on polyhydroxyl reagents, using the overall reaction system reactivity evaluation by MALDI MS.

## Figures and Tables

**Table 1:** Parameters of the PEG-IPDI reaction and measured reaction rates.

# Sample	Temperature (°C)	%wt ^1^	Molar Ratio PEG/IPDI/DBTL	*k_app_* (min^−1^)
1	35	15	1/2/0.018	1.13 × 10^−2^
2	35	30	1/2/0.018	3.81 × 10^−2^
3	35	5	1/2/0.018	0.29 × 10^−2^
4	35	15	1/2/0.054	1.59 × 10^−2^
5	50	15	1/2/0.018	2.75 × 10^−2^
6	25	15	1/2/0.018	0.09 × 10^−2^
7	35	15	1/2/0	0

^1^ Calculated as reactants content (IPDI, PEG, and DBTL) in the reaction mixture.

**Table 2:** The equations used for the structural assignment of the MALDI MS monoisotopic peaks correspond to each type of series for the samples analyzed with DCTB.

Series	*m*/*z*
*P*0	*44*× *n (ethylene oxide units) + 18 (end groups of PEG) + 23 (Na^+^)*
*P*1′	*44* × *n (ethylene oxide units) + 18 (end groups of PEG) + 23 (Na^+^) + 222 (IPDI)*
*P*2′	*44* × *n (ethylene oxide units) + 18 (end groups of PEG) + 23 (Na^+^) + 2* × *222 (IPDI)*

**Table 3:** The equations used for the structural assignment of the MALDI MS monoisotopic peaks correspond to each type of series for the samples analyzed with DHB.

Series	*m*/*z*
*P*0	44 × *n* (*ethylene oxide units*) *+* 18 (*end groups of PEG*) *+* 23 (*Na^+^*)
*P*1	44 × *n* (*ethylene oxide units*) *+* 18 (*end groups of PEG*) *+* 23 (*Na^+^*) *+* 222 (*IPDI*) *+* 32 (*MeOH*)
*P*2	44 × *n* (*ethylene oxide units*) *+* 18 (*end groups of PEG*) *+* 23 (*Na^+^*) *+* 2 × 222 (*IPDI*) *+* 2 × 32 (*MeOH*)

## Data Availability

Not applicable.

## Supplementary Materials

The following supporting information can be downloaded at: https://www.mdpi.com/article/10.3390/ma16020821/s1, Figure S1: MS/MS fragmentation spectrum of the [PEG + IPDI + MeOH + Na]^+^ adduct ion with 47 ethylene oxide monomer units of (*m*/*z* = 2362.5); Scheme S1: Fragmentation pathways of the [PEG + IPDI + MeOH + Na]^+^ adduct ion; Figure S2: MS/MS fragmentation spectrum of the [PEG + 2IPDI + 2MeOH + Na]^+^ adduct ion with 47 repeating units (*m*/*z* = 2616.6); Scheme S2: Fragmentation pathways of the [PEG + 2IPDI + 2MeOH + Na]^+^ adduct ion; Figure S3: *k_app_* determination from the evolution of *ln*([*P*0]_0_/[*P*0*]_t_*) in time.
