# Supplementary material for: Polyethylene Glycol-Isophorone Diisocyanate Polyurethane Prepolymers Tailored Using MALDI MS
Source: Materials (Basel). 2023 Jan 14;16(2):821. doi: 10.3390/ma16020821 (PMC9862538; doi:10.3390/ma16020821)
Supplement: Supplementary file 1 [file materials-16-00821-s001.zip › materials-2122398-supplementary.pdf]

materials

Supplementary Information

Polyethylene Glycol-Isophorone Diisocyanate Polyurethane Prepolymers Tailored Using MALDI MS

Diana-Andreea Blaj, Alexandra-Diana Diaconu, Valeria Harabagiu and Cristian Peptu *

"Petru Poni" Institute of Macromolecular Chemistry, Grigore Ghica Voda Alley, 41A, 700487 Iasi, Romania
* Correspondence: cristian.peptu@icmpp.ro

Contents

MS/MS studies (Figures S1, S2, Schemes S1, S2) – pages S1-S4
Determination of apparent reaction rates for synthesis performed at different temperatures (Figure S3) – page S4

MS/MS studies

The fragmentation mass spectrum of the methanol (MeOH) quenched mono-functional PEG $[PEG+IPDI+MeOH+Na]^{+}$having 47 ethylene oxide constitutional units,parent ion found at$m/z = 2363$ (*P1* series), is presented in Figure S1. The MS/MS spectrum revealed the corresponding fragment ions and neutral losses resulting from the main fragmentation pathways, the cleavage of the urethane bonds, and the cleavage of the ether bonds of the PEG chain, as shown in Scheme S1. The cleavage of the end-chain urethane bond on the acyl side leads to methanol neutral loss. Moreover, the cleavage on the acyl side of the secondary urethane bond leads to a highly intense daughter ion (m/z = 2108) with an associated neutral loss of 254 Da corresponding to amethanol-modified IPDI structure.The cleavage of the urethane bond on the alkyl side leads to a neutral loss of 272 Da. On the other hand, the cleavage of the PEG chain results in consecutive neural losses of 44 Da, corresponding to the ethylene oxide units, from either the IPDI functionalized end, or from the hydroxyl end-chain, whose structures are presented in Scheme S1.

Citation: Blaj, D.-A.; Diaconu, A.-D.; Harabagiu, V.; Peptu, C. Polyethylene Glycol-Isophorone Diisocyanate Polyurethane Prepolymers Tailored Using MALDI MS. *Materials* **2023**, 16, 821. https://doi.org/10.3390/ma16020821

Academic Editor: Rafał Frański

Received: 10 December 2022
Revised: 11 January 2023
Accepted: 12 January 2023
Published: 14 January 2023

Copyright: © 2023 by the authors. Submitted for possible open access publication under the terms and conditions of the Creative Commons Attribution (CC BY) license (https://creativecommons.org/licenses/by/4.0/).

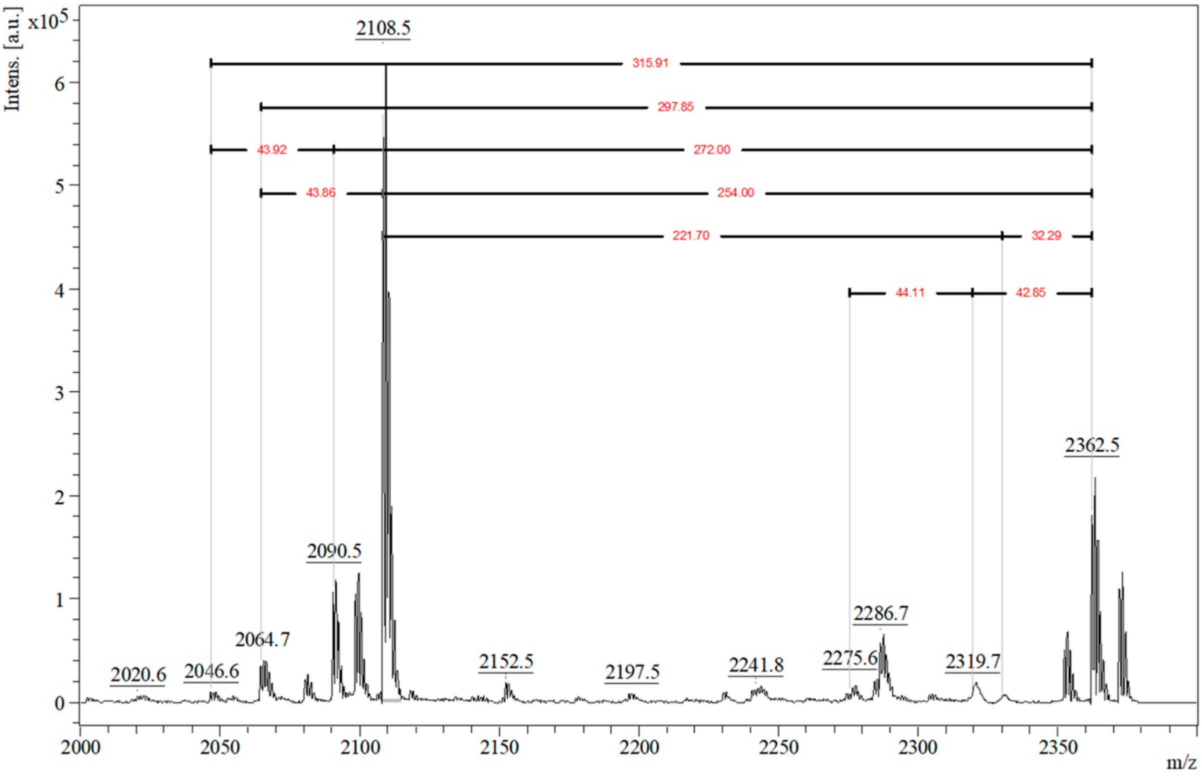

Figure S1. MS/MS fragmentation spectrum of the [PEG+IPDI+MeOH+Na]+ adduct ion with 47 ethylene oxide monomer units of (*m*/*z* = 2362.5).

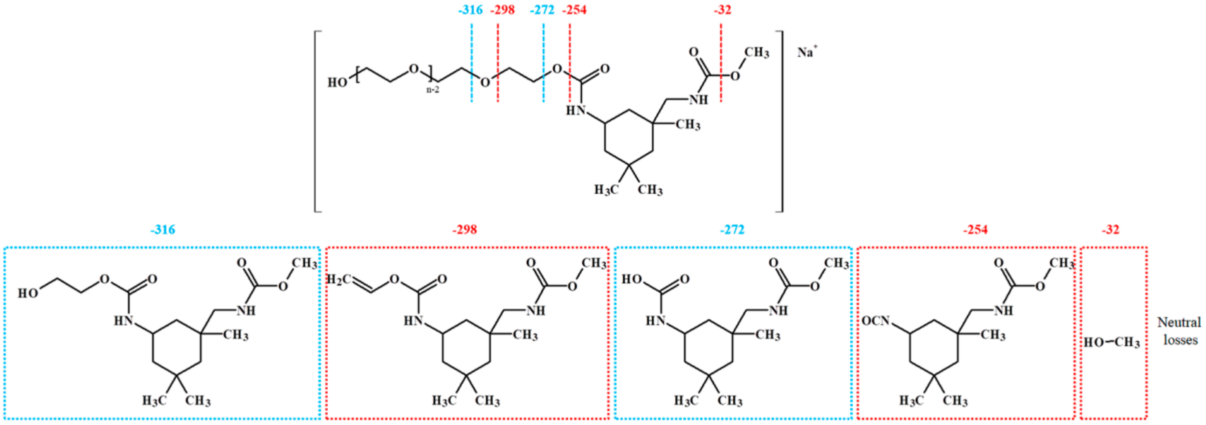

Scheme S1. Fragmentation pathways of the [PEG+IPDI+MeOH+Na]+ adduct ion.

The MS/MS fragmentation was also performed for the quenched di-functional PEG [PEG+2xIPDI+2xMeOH+Na] + parent ion found at *m*/*z* 2618, corresponding to 47 ethylene oxide monomer units that belong to the *P2* series. The MS/MS mass spectrum is shown in Figure S2, while the fragmentation pathways are presented in Scheme S2. Similar to the *P1* series, two main fragmentation pathways are observed: cleavage of the urethane bonds and cleavage of the PEG chain, the latter resulting in 44 Da neutral losses. As expected from a di-functional structure, an additional neutral loss of 254 Dais present in the fragmentation spectrum, as compared with the fragmentation of P1 species. Thus, besides the fragment peak at m/z=2362 corresponding to the neutral loss of one MeOH-modified IPDI molecule, the MS/MS spectrum presents an additional peak at m/z=2108, associated with the cleavage of another MeOH-modified IPDI molecule. Moreover, the neural losses of 272, 298, and 316 Da could also be identified in the fragmentation spectrum of the parent ion coming fromthe P2 series, fragmentation processes being described in Scheme S2.

Therefore, the MS/MS fragmentation spectra of peaks associated with *P1* and *P2*species confirm their structural assignment.

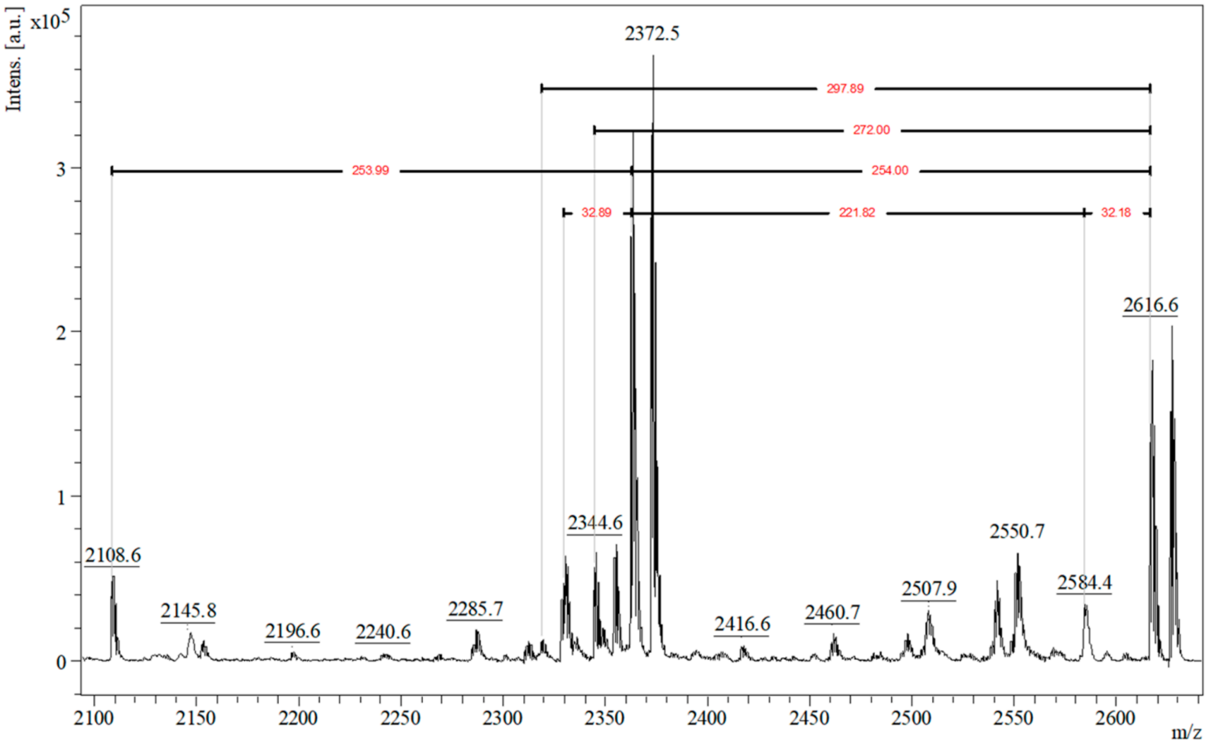

Figure S2. MS/MS fragmentation spectrum of the [PEG+2IPDI+2MeOH+Na]+ adduct ion with 47 repeating units (*m*/*z* = 2616.6).

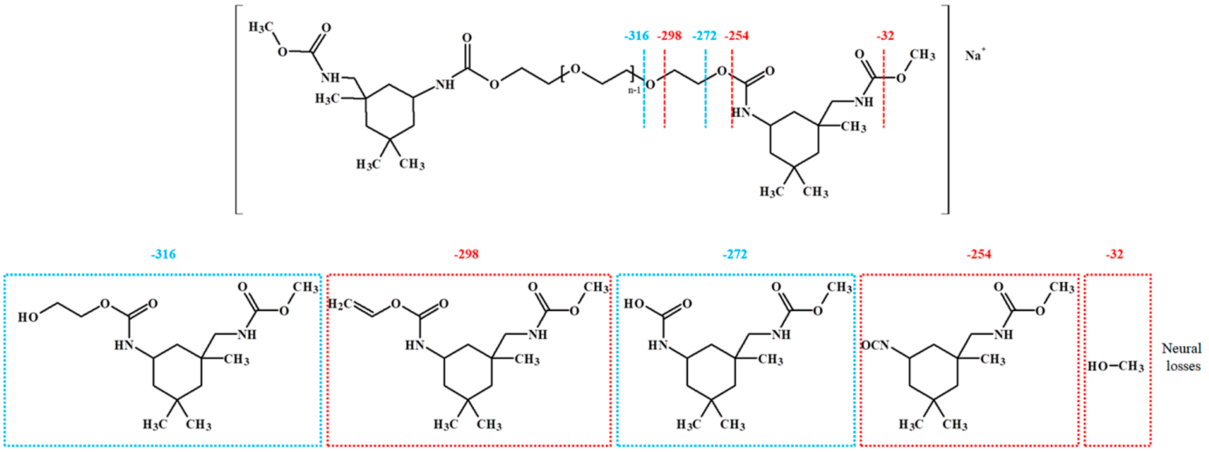

Scheme S2. Fragmentation pathways of the [PEG+2IPDI+2MeOH+Na]+ adduct ion.

Determination of apparent reaction rates (k_{app}) for synthesis performed at different temperatures

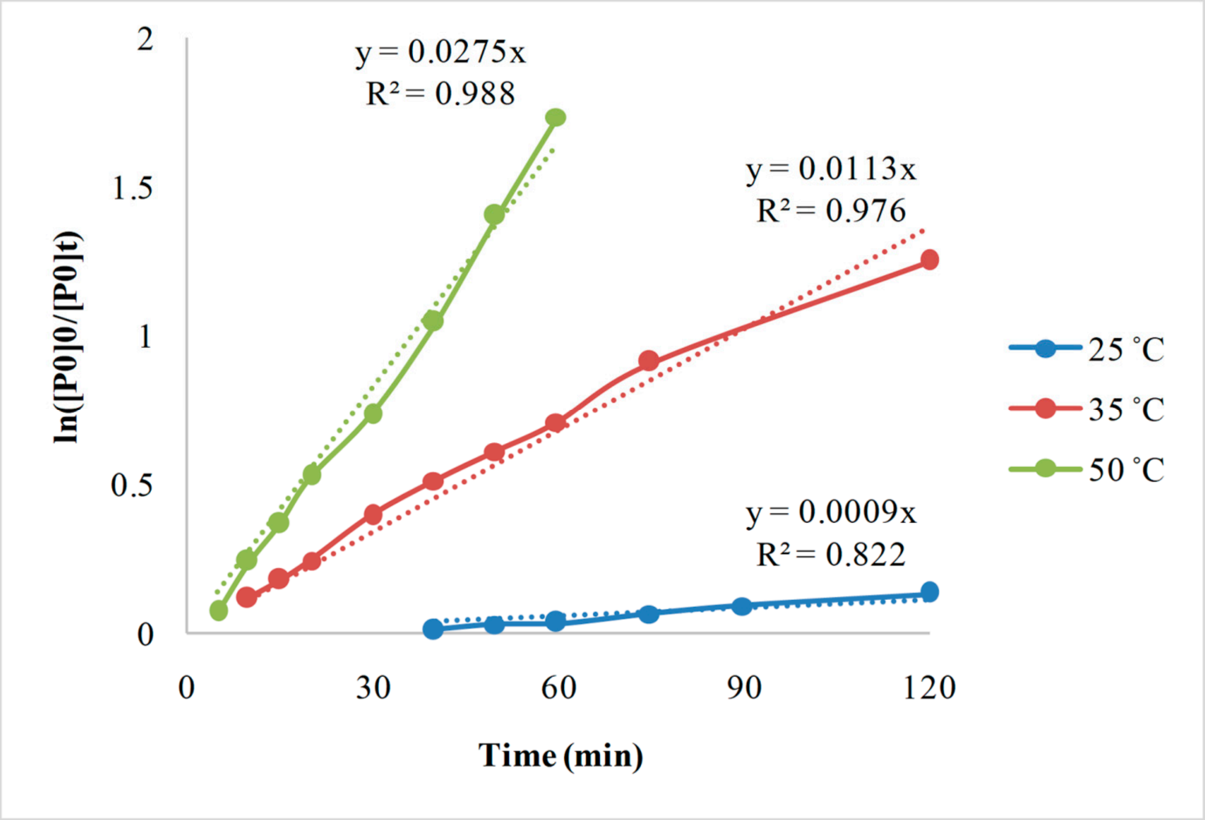

Figure S3. K_{app} determination from the evolution of *ln([P0]*$_0$*/[P0]*$_t$*)* in time.
